# Differential Effects of Dietary MSG on Hippocampal Dependent Memory Are Mediated by Diet

**DOI:** 10.3389/fnins.2019.00968

**Published:** 2019-09-12

**Authors:** Kathleen F. Holton, Sara L. Hargrave, Terry L. Davidson

**Affiliations:** ^1^Nutritional Neuroscience Laboratory, Department of Health Studies, Center for Behavioral Neuroscience, American University, Washington, DC, United States; ^2^National Cancer Institute, National Institutes of Health, Rockville, MD, United States; ^3^Laboratory for Behavioral and Neural Homeostasis, Department of Psychology, Center for Behavioral Neuroscience, American University, Washington, DC, United States

**Keywords:** glutamate, MSG, diet, western diet, obesity, memory, hippocampus

## Abstract

**Introduction:**

Free glutamate is a common dietary flavor enhancer and is also an important excitatory neurotransmitter in the body. A good number of food additives which contain glutamate are found in the Western Diet, and this diet has also been linked to increased risk of cognitive dysfunction.

**Objective:**

To examine the effects of dietary glutamate on hippocampal and non-hippocampal memory performance, and whether consuming a diet high in fat/sugar could influence any observed associations.

**Methods:**

Sixty-four adult male Sprague-Dawley rats were trained concurrently on two different discrimination problems: (1) Pavlovian serial feature negative (sFN) discrimination, in which a brief tone stimulus was reinforced with sucrose pellets when it was presented alone (T+ trials) and non-reinforced on trials when it was preceded by the presentation of a brief light (LT− trials); and (2) a simple discrimination (SD) problem in which a white noise (WN+) cue was reinforced with sucrose pellets and a clicker (C-) stimulus was not reinforced. Previous research has shown that sFN, but not SD performance, depends on the functional integrity of the hippocampus. After solving both problems, the rats were assigned to one of four *ad libitum*-fed diet groups, matched on weight and discrimination performance: (1) high fat, high sugar western-style diet (WD), (2) standard laboratory rodent chow diet (chow), (3) WD + monosodium glutamate (MSG), or (4) chow + MSG.

**Results:**

After 14 weeks, rats fed WD had higher adiposity than rats fed chow. Consistent with previous findings, rats fed WD exhibited impaired performance on the sFN problem, but not on the SD, relative to rats fed chow. Adding MSG to WD abolished this impairment, whereas rats fed chow + MSG had impaired sFN performance compared to rats fed chow alone. No differences in performance on the SD task were observed.

**Conclusion:**

This study demonstrates differing effects of dietary glutamate on hippocampal dependent memory function, with MSG impairing hippocampal function in animals receiving chow, while improving hippocampal function in animals receiving a Western-type diet, high in fat and sugar. More research will be needed to explore the cause of these differential effects.

## Introduction

According to the 2015 Dietary Guidelines Advisory Committee Scientific Report (Millen et al., 2016), the overall United States population consumes higher than recommended amounts of saturated fat, refined grains and sugar, and lower than recommended amounts of vegetables, fruits, whole grains, and dairy. This general pattern of intake, often referred to as the Western dietary pattern, has become increasingly popular not only in the United States, but in many other Western and westernized countries (Imamura et al., 2015). A variety of evidence links Western dietary pattern to increases in body weight, adiposity, Type II diabetes, and higher incidence of cardiovascular and neurodegenerative diseases, among others (for reviews see Rodriguez-Monforte et al., 2017; Medina-Remon et al., 2018).

Rats and humans maintained on western-style diet (WD) have also been reported to exhibit deficits in several types of hippocampal-dependent memory, and these deficits are accompanied by several signs of hippocampal pathophysiology (Stranahan, 2015; Yeomans, 2017; Davidson et al., 2019). The hippocampus has been shown to be necessary for learning relationships among objects in space (i.e., spatial memory) in animals (Forwood et al., 2005) and humans (Goodrich-Hunsaker and Hopkins, 2010). In addition, both species have difficulty retrieving memories of certain types of past experiences (i.e., episodic memory) following interference with hippocampal functioning (Dede et al., 2016; Drieskens et al., 2017); and both rats (Moses et al., 2005) and humans (Konkel et al., 2008) are impaired in forming or utilizing certain types of relationships among items in memory (relational memory) when hippocampal function is disrupted.

Rats and other preclinical models have provided important information about the potentially harmful effects of WD consumption on hippocampal-dependent memory and cognitive processes. However, one problematic aspect of preclinical studies is that the WD used with rats typically fails to recapitulate a number of potentially important characteristics of the human Western dietary pattern (Hintze et al., 2018). For example, the foods that comprise the human Western dietary pattern often contain food additives such as free glutamate, which serves to stimulate neurons on the tongue, thereby enhancing the flavor of the food (Beauchamp and Mennella, 2009). Typically, the blood–brain barrier is thought to protect humans from excess amounts of dietary glutamate accessing the brain (Smith, 2000); however, there are individuals with multi-symptom illness, including cognitive dysfunction, who have symptom remission from the removal of dietary glutamate, with return of symptoms upon challenge with glutamate, relative to placebo, in a double-blind placebo-controlled fashion (Holton et al., 2012). This suggests that dietary glutamate may be able to more freely pass the blood–brain barrier in a proportion of the population.

Free glutamate is not a normal component of the WD or chow diets typically given to rats. This omission could be very important because one potential mechanism for memory/learning impairment is altered glutamatergic neurotransmission in the hippocampus. It is well known that normal glutamatergic signaling is necessary for memory formation and learning (Baez et al., 2018). Abnormal handling of glutamate (leading to excitotoxicity), or altered expression of glutamate receptors, could potentially lead to impaired hippocampal function (Brigman et al., 2010; Huo et al., 2015). On the other hand, dietary glutamate could potentially improve performance if glucose is in low supply, such as in metabolic syndrome or diabetes, by providing a substrate which can be converted to alpha-ketoglutarate to support ATP production in the mitochondria (Dienel, 2013).

The objective of the present study was to examine the effects of dietary glutamate on hippocampal and non-hippocampal dependent memory performance in Sprague-Dawley rats and whether consuming a diet high in fat/sugar can influence any observed associations.

## Materials and Methods

### Background on Methods Used

Previous research has shown that rats with selective neurotoxic lesions of the hippocampus are impaired in the ability to solve a serial feature negative (sFN) discrimination problem, which requires them to learn that the presentation of one stimulus (e.g., a brief light) signals that another stimulus (e.g., a brief tone) will *not* be followed by the delivery of a food. On trials during which no light is presented, the tone is followed by food (Holland et al., 1999). In contrast to the impairment on the sFN task, hippocampal lesions have little to no effect on the performance of a simple discrimination (SD) problem that requires the rats to learn that one stimulus (e.g., a clicker) is followed by food and another stimulus (i.e., a white noise) is not reinforced. This SD task only requires that rats learn the simpler relationship between the clicker and food. This learning does not depend on the hippocampus because uncertainty resolution is not required. Differentially, the sFN problem requires that rats use the light to resolve ambiguity or uncertainty in the relationship between the presentation of the tone and food. Evidence from studies of animals and humans indicates that this type of ambiguity resolution is hippocampal-dependent; for examples of this please see Barense et al. (2005) and Vanni-Mercier et al. (2009).

Like rats with hippocampal lesions, rats maintained on WD are also impaired at solving the sFN problem relative to control rats fed standard chow, but are not impaired relative to chow controls in solving the SD problem (Kanoski et al., 2010; Davidson et al., 2012, 2013). This pattern of results indicates that a hippocampal-dependent learning memory process has been affected (Holland et al., 1999; Davidson et al., 2014; Sakimoto and Sakata, 2018). Therefore, the present study uses the sFN and SD problems to differentiate hippocampal versus non-hippocampal effects.

### Timeline

**FIGURE 1:**
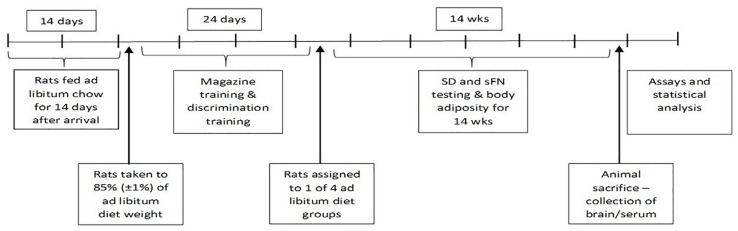
Timeline of study procedures.

A timeline for the study procedures is included in Figure 1.

### Subjects

Sixty-four experimentally naïve, male Sprague-Dawley rats, approximately 90 days of age and weighing 350–375 g, were purchased from Envigo (Indianapolis, IN, United States). It was identified in week 10 that two animals inadvertently had their diets switched, so the data for these two animals were dropped from the analyses, leaving 15 animals in each of the WD and Chow + MSG groups, and 16 animals in the Chow and WD + MSG groups), or 62 animals in total. All of these rats were used in all experiments described below, except for the serum and tissue glutamate analyses, where one animal’s data was lost. The animals were housed individually in a climate-controlled environment under a 14:10 h light:dark cycle with the light phase beginning at 0900 h each day. The rats had free-access to water throughout the study except during experimental sessions as described below. The care and use of all animals in this study was reviewed and approved by the American University Institutional Animal Care and Use Committee.

### Apparatus

All training and testing sessions were conducted in 16 identical conditioning chambers constructed of aluminum end walls and Plexiglas sidewalls, measuring 59.7 × 34.3 × 26.35 cm (Lafayette Instruments). The floors of the chamber consisted of stainless-steel metal rods measuring 0.48 cm in diameter and 1.07 cm apart. A recessed food magazine was in the center of one end wall of each chamber. A motorized pellet dispenser attached to each chamber was used to deliver 45-mg sucrose pellets (Research Diets, Lancaster, NH, United States) to the food magazine. Entries into the food magazine were recorded by a computer-operated infrared monitoring system. One infrared photo transmitter and one receiver were located on each side wall of the recessed food magazine such that rats would break a photobeam to gain entry to the magazine. Diffuse tone (1500 Hz), white noise and clicker (3 Hz) served as auditory stimuli (all approximately 76–78 db). A 6-watt light conditioned stimulus measuring 2.4 cm in diameter was located 5 cm to the left of and 6 cm above the recessed food magazine. Each chamber was housed in a ventilated sound-attenuating enclosure. A computer located in an adjoining room controlled the timing and presentation of all programed experimental events.

### Diets

Four diets were used in this study. (1) Western diet (WD) was high in saturated fat and dextrose (Harlan Teklad, Indianapolis, IN, United States TD.04489) and contained the following ingredients (g/kg): 270 g casein, 220.5 g glucose, 200 g cornstarch, 170 g lard, 50 g cellulose, and 15 g safflower oil. This diet had a caloric density of 4.5 kcal/g and contained the following percentages of energy from the three macronutrient classes: 38% kcal from carbohydrate, 21% kcal from protein, and 40% kcal from fat. (2) Standard laboratory rodent chow diet (Lab Diets 5001) was used for the control diet (CHOW). This control diet had a caloric density of 3.0 kcal/g and contained the following percentages of energy from the three macronutrient classes: 60% kcal from carbohydrates, 28% kcal from protein, and 12% kcal from fat. (3) WD + MSG was the same as the WD described above with 0.4% MSG/kcal of monosodium glutamate (MSG) added. (4) The CHOW + MSG also had MSG added at 0.4% MSG/kcal. This amount of MSG was chosen based on the recommendations from the glutamate association on the optimal percentage of MSG needed for flavor enhancing effects, which taste panel studies reported to be 0.1–0.5% MSG by weight of food (Miyaki et al., 2016). MSG was added on a kilocalorie basis instead of a gram basis, since rats receiving the WD diet tend to eat less total grams of food (but more total calories) than the animals receiving chow.

### Procedure

The procedures used for magazine and discrimination training followed those described previously (Davidson et al., 2012). The rats were fed standard rodent laboratory chow *ad libitum* for 14 days after their arrival in the laboratory, after which food rationing was used to gradually reduce the body weight of each rat to 85% of the average weight obtained on the last 2 days of *ad libitum* feeding. Behavioral training began when all rats achieved this 85% (±1%) body weight criterion. The rats were weighed immediately prior to each of these initial training and probe test sessions. All rats received their daily food ration immediately after the completion of each session under food deprivation.

### Magazine Training

The rats were assigned to four squads, of 16 rats each, with each rat assigned to one of the 16 conditioning chambers for the duration of the study. All rats then received one 10-min session of magazine training to habituate them to the apparatus and to learn the location of the food cup. During this session, 10 presentations of two sucrose pellets were delivered according to a variable-time 60-s schedule (i.e., average of one presentation of two pellets per min).

### Discrimination Training

All rats were given one 60-min session of discrimination training per day for 24 consecutive days while continuing to follow the chow diet. These sessions were conducted between 0930 and 1400 h. In each session, the rats were trained concurrently on sFN and SDs. For *serial feature negative discrimination training* (sFN), rats were given trials in which the presentation of a 5-s tone (T) terminated with the delivery of two sucrose pellets (on T+ trials); whereas no pellets were delivered after a 5-s illumination of the panel light (L) followed by a 5-s empty interval (i.e., with no programed stimulus) preceded presentation of the 5-s tone (on L→T− trials). For *simple discrimination training* (WN+/C-), a 5-s presentation of white noise (WN) terminated with the delivery of two sucrose pellets on each rewarded (WN+) trial; whereas no pellets were delivered after a 5-s presentation of the clicker (C-) on non-rewarded trials. Each session consisted of one T+ trial and three L→T- trials along with one WN+ trial and three C- trials. Trial orders were randomized for each session, and the inter-trial interval varied within the range of 300–600 s (average = 450 s).

### Discrimination Probe Testing

After asymptotic discrimination training was achieved, the rats were assigned to four diet groups of 16 rats each that were matched in terms of mean (SD) body weight and discrimination performance on both the sFN (LT−, T+) and SD tasks (WN+, C−) based on what was recorded over the last two sessions of acquisition training. The four diet groups were: (1) Western Diet (WD), (2) Chow, (3) WD + MSG, and (4) Chow + MSG. The discrimination performance, body weight, and body adiposity of all groups was assessed at the end of 1, 2, 3, 4, 6, 8, 10, 12, and 14 weeks of *ad libitum* feeding of their designated diets. On each of these weeks all groups were tested concurrently with both the SD and sFN discrimination problems in two consecutive sessions that were conducted using the same parameters as described during original training. On each week, body weights and body adiposity were recorded on the day after the last behavioral test session. Body fat percentage of each rat was measured using an EchoMRI-900 magnetic resonance body composition analyzer (Echo Medical Systems, LLC, Houston, TX, United States).

### Sacrifice and Sample Collection

At the completion of probe testing, rats were anesthetized via isoflurane inhalation and rapidly decapitated with a guillotine. Trunk blood was collected into K3EDTA + tubes and temporarily stored on ice. Brains were then rapidly rinsed in 1X phosphate buffered saline (PBS), hippocampi and hypothalami were dissected on ice, placed into nuclease-free microcentrifuge tubes, and stored on dry ice for the duration of sacrifice.

### Protocol for Dissecting the Hypothalamus

On the ventral side of the rat brain, the hypothalamus was delimited by the optic chiasm and the anterior commissure, and then was easily visualized using a surgical microscope. Dissection was accomplished by gently pushing down then pinching around the hypothalamus using curved forceps. The forceps were then used to carefully lift the hypothalamus out of the brain.

### Protocol for Dissecting the Hippocampus

Using a sharp razor blade or scalpel, the cortex was opened at the midline and small forceps were then inserted and opened to separate the two hemispheres. Working on one hemisphere at a time, the hemisphere was placed on its side in order to reveal the midsagittal section of the brain. Closed curved forceps were inserted right above the rostral part of the corpus callosum, then gently opened and closed repeatedly alongside the corpus callosum in order to separate it from the cortex above. This revealed the dorsal part of the hippocampus, located deeper behind the corpus callosum. Once the hippocampus was visible, it was gently rolled out of the brain using the closed curved forceps. These steps were then repeated for the remaining hemisphere.

### Serum and Tissue Analyses

All samples were stored at −80°C until analysis was performed. L-glutamate levels were assessed in blood serum and hippocampal and hypothalamic tissue homogenates using a commercially available assay (K-GLUT, Megazyme, Inc.) (Beutler, 1990) using the Microplate Assay Procedure. First, tissue homogenates were deproteinated using 1 M perchloric acid (4× tissue volume), centrifuged at 1500 × *g* for 10 min, then neutralized with potassium hydroxide. Total volume of liquid was noted so that small variances in dilution did not affect the expressed values of glutamate in tissue. After deproteinization and neutralization, sample supernatants, as well as blanks and standards (50 and 100%) were pipetted into 96-well plates (with separate blanks and standards for each plate). Buffer, NAD+/INT and diaphorase were also added to wells and mixed. Initial absorbance was measured after 2 min, then GIDH was added to the wells, which were read every 2 min until the absorbance plateaued. The change in absorbance for the blank wells was subtracted from all other wells, then each sample was divided by the absorbance of the 100% standard and multiplied by the dilution factor of each well. The 50% standard was used to check each plate for accuracy; plates were between 47 and 53% of the 100% standard. Serum samples were not deproteinated, however, they were otherwise run using the same method as the tissue samples.

### Data Analysis

Normality of the data was confirmed using Kolmogorov-Smirnov tests. Data from training and probe testing were evaluated using multi-factor analysis of variance (ANOVA). The dependent variable was the mean number of beam breaks. Pre-test baseline weight obtained at the completion of training (PRE; 85% of original *ad libitum* weight), and weight prior to each probe test (1–9) during the 14-week test phase, were within-subjects factors, with Diet (WD and Chow) and MSG exposure (MSG or no MSG) as between-subjects factors. Diet and MSG exposure were also used as between-subject factors for ANOVA of terminal body adiposity and serum MSG levels after the end of testing. The data from the discrimination training phase were evaluated using Problem Type (sFN or SD), ± [rewarded (+) vs. non-rewarded (−) trials], Blocks (12 blocks of training sessions) and Sessions (2 sessions per block) as within-subjects factors, and Diet (WD and Chow) and MSG exposure (MSG or no MSG) as dummy between-subjects variables. The ANOVA for probe testing included the same within- and between-subject factors, except that Probe test (1–9) was used as a within-subjects variable. Pairwise comparisons using *post hoc* Dunnett tests were used to evaluate significant interactions between groups and main effects as needed. In addition, difference scores calculated by subtracting the number of beam breaks recorded on “−” trials from those recorded on “+” trials during each probe test were used to assess the magnitude of discrimination differences when significant interactions involving Diet, MSG exposure, and rewarded vs. non-rewarded trials (±) were obtained. Alpha level for all comparisons was set at *p* = 0.05. Statistica 13 Ultimate Academic Bundle software 2018 (Palo Alto, CA, United States) was used for all statistical analyses.

## Results

### Body Weight and Adiposity

**FIGURE 2:**
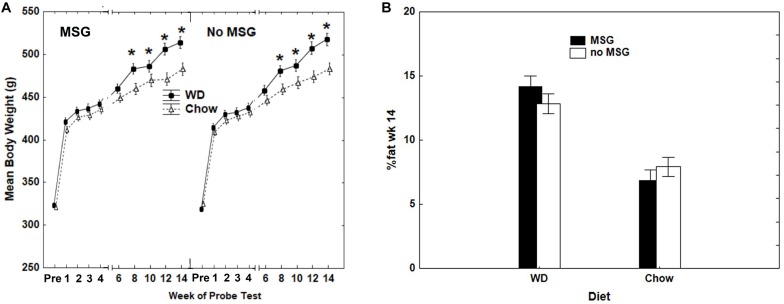
**(A)** Differences in mean body weight as a function of Diet and MSG exposure prior to probe testing, when training was completed at 85% of original *ad libitum* weight (PRE), and for the weeks that probe testing was completed during the 14-week period of *ad libitum* feeding. *N* = 62 animals. **(B)** Body adiposity (percent body fat) after the completion of 14 weeks of probe testing as a function of Diet (Chow or WD) and MSG exposure. *N* = 62 animals.

Rats fed the WD gained significantly more body weight than rats fed Chow. At the end of 14 weeks, rats fed WD showed a 60.7% (SEM = ±0.016) weight gain relative to their pre-diet baseline, whereas body weight for chow-fed controls increased by 49.5% (SEM = ±0.009) compared to their pre-diet baseline. Using Diet and MSG exposure as between-subjects factors, resulted in a significant main effect of Diet (*p* < 0.001), but no main effect or interaction involving MSG was significant. Thus, weight gain did not vary based on MSG exposure for either diet. Figure 2A depicts body weight for both diet and MSG groups, beginning at the end of acquisition training (pre-test diets) and throughout the 14-week test phase (while on the test diets). Significant main effects were observed for Diet (WD compared to Chow) (*p* < 0.01), Test Weeks (*p* < 0.01) and a significant interaction was observed between Diet and Test Weeks (*p* < 0.01). No main effect of MSG, nor interaction, achieved significance. As expected, rats fed WD had higher adiposity compared to rats consuming standard chow. Figure 2B shows that the two groups of rats maintained on WD had a higher percent body fat compared to the two groups of rats maintained on Chow at the end of 14 weeks of *ad libitum* feeding. The effect of Diet on adiposity was significant, with the two groups of animals receiving WD having significantly greater adiposity than the two groups receiving Chow (*p* < 0.01), whereas neither the main effect of MSG treatment, nor the interaction of that factor with Diet, were significant. Thus, similar to body weight, MSG did not appear to significantly influence body adiposity.

### Serial Feature Negative Discrimination and Simple Discrimination

#### Training

**FIGURE 3:**
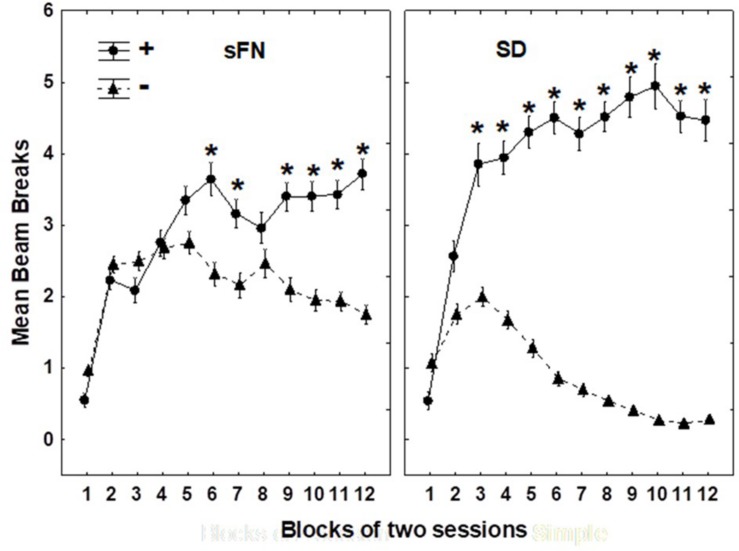
Hippocampal dependent serial feature negative (sFN) and non-hippocampal dependent simple discrimination (SD) performance on each two-session block of training. ^∗^Indicate a significant difference between circles, which are rewarded (+) trials, and the triangles, which are non-rewarded (−) blocks. *N* = 62 animals.

Figure 3 shows that all rats solved both the sFN and SD problems by the end of 12 two-session blocks of training with each type of problem. Significant main effects were observed for Blocks (*p* < 0.01) and for rewarded vs. non-rewarded trials (*p* < 0.01), as well as a significant interaction between rewarded vs. non-rewarded trials and Blocks (*p* < 0.01), rewarded vs. non-rewarded trials and Problem Type (*p* < 0.001), and interactions between rewarded vs. non-rewarded trials, Blocks, and Problem Type (*p* < 0.01).

Although the rewarded vs. non-rewarded trials were significant for both the sFN and SD discrimination problems based on training, the magnitude of this difference was greater for the SD compared to the sFN discrimination. Therefore, we analyzed the effects of Diet and MSG during probe testing separately for each test.

#### Serial Feature Negative Probe Testing

**FIGURE 4:**
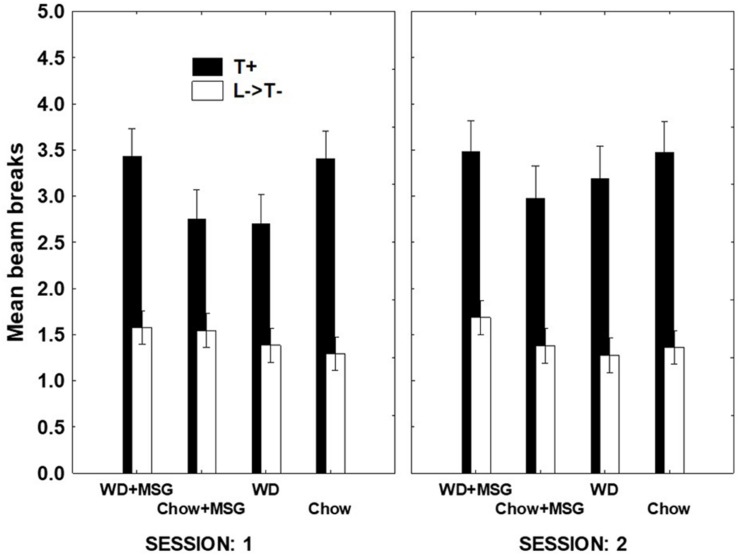
Hippocampal dependent serial feature negative (sFN) discrimination for each treatment condition as a function of session during probe testing. T+ means a tone was sounded and reward was given. L→ T– means a light signaled that the tone would not be rewarded. Greater distance between black and white bars signals better discrimination performance. *N* = 62 animals.

For the sFN discrimination, Figure 4 shows that while the same pattern of findings appeared in both sessions, the magnitude of differences due to Diet and MSG exposure was larger on Session 1 compared to Session 2. This finding was confirmed, yielding a significant interaction between Session, rewarded vs. non-rewarded trials, Diet, and MSG exposure (*p* < 0.05). A separate analysis of the data from Session 1 yielded a significant main effect of rewarded vs. non-rewarded trials (*p* < 0.01) and more importantly, a significant interaction between these trials with Diet and MSG exposure (*p* < 0.05). This interaction did not vary by Probe Test, nor did Diet or MSG Treatment interact significantly with rewarded vs. non-rewarded trials on their own. The effect of Session on sFN performance may have occurred because performance on the first session of each probe test depends more on long-term memory compared to the second session.

**FIGURE 5:**
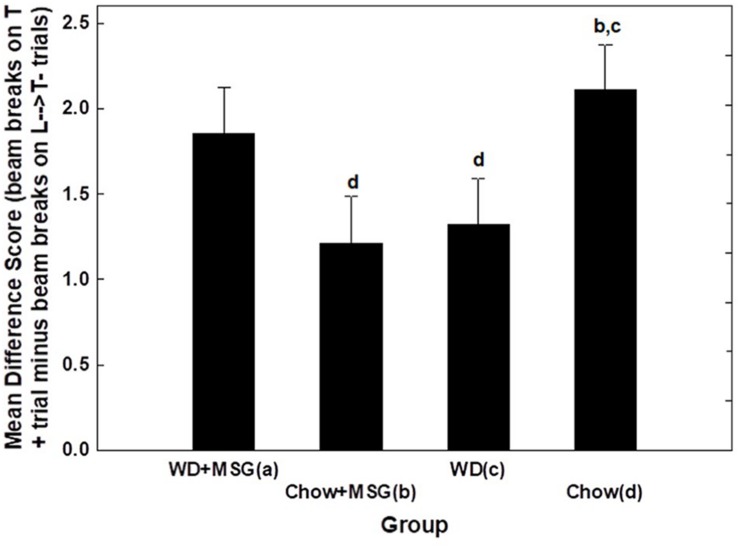
Difference scores comparing the magnitude of hippocampal dependent serial feature negative (sFN) discrimination performance among diet groups (session 1) during probe testing. Letters in the figure indicate significant pairwise comparisons (*p* < 0.05). *N* = 62 animals.

To further evaluate the interacting effects of Diet and MSG on sFN performance during Session 1, we calculated difference scores by subtracting the beam breaks recorded on non-rewarded trials from those recorded on rewarded trials. We then analyzed these difference scores using Diet, MSG exposure and Probe Test as independent variables (see Figure 5). This analysis yielded a significant interaction between Diet and MSG exposure (*p* < 0.01) that did not vary significantly as a function of Probe Test. *Post hoc* Dunnett tests were used to compare each of the Diet and MSG conditions to Chow as the control group. For rats given WD alone, sFN performance was impaired relative to Chow (*p* < 0.04). However, sFN discrimination performance for rats that received WD + MSG was higher, with these rats performing most similarly to the Chow group. In contrast, discrimination performance for rats that received Chow + MSG was significantly lower than the performance of rats that received Chow alone (*p* < 0.02).

#### Simple Discrimination Testing

**FIGURE 6:**
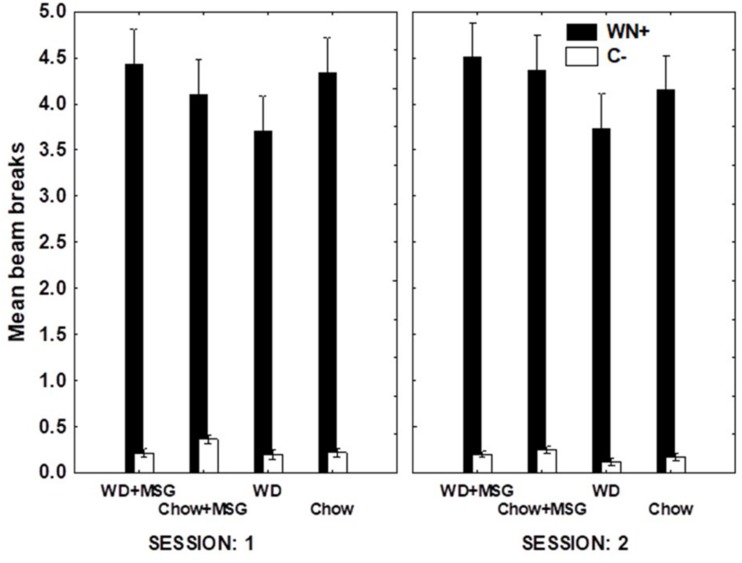
Non-hippocampal dependent simple discrimination (SD) performance for each condition as a function of session during probe testing. WN+ means animals were rewarded after a white noise sound, while C- means animals were not rewarded after a clicker sound. Main effect of rewarded vs. non-rewarded trials was significant (*p* < 0.01), but pairwise comparisons were non-significant. *N* = 62 animals.

**FIGURE 7:**
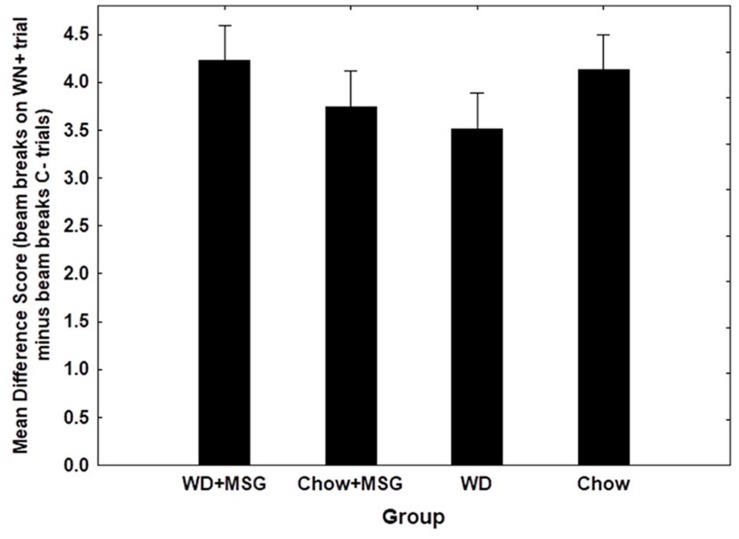
Difference scores comparing the magnitude of non-hippocampal dependent simple discrimination (SD) performance among groups (Session 1). No significant effects observed. *N* = 62 animals.

Figure 6 shows overall test performance on the SD problem as a function of Session, rewarded vs. non-rewarded trials, Diet, and MSG exposure. The main effect of the rewarded vs. non-rewarded trials was significant (*p* < 0.01); however, neither the interaction of these with the session number, nor the further interaction with Diet and MSG exposure achieved significance. Furthermore, no significant main effects of Diet or MSG were observed, and no interactions involving either or both factors combined with rewarded versus non-rewarded trials were significant. Although there was no significant effect of Session for the SD condition, the magnitude of the difference among the various groups appeared somewhat larger on Session 1 compared to Session 2. Because of this, we used an analysis of difference scores, like that used for the sFN discrimination, to compare the effects of Diet and MSG on SD performance on Session 1. Figure 7 shows that the pattern of relative differences between each of the treatment conditions was similar to that observed for the sFN discrimination. However, unlike sFN discrimination performance, differences between the groups were not significant. No significant main effects of Diet or MSG were observed, nor was there a significant Diet by MSG interaction.

### Serum and Tissue Glutamate

**FIGURE 8:**
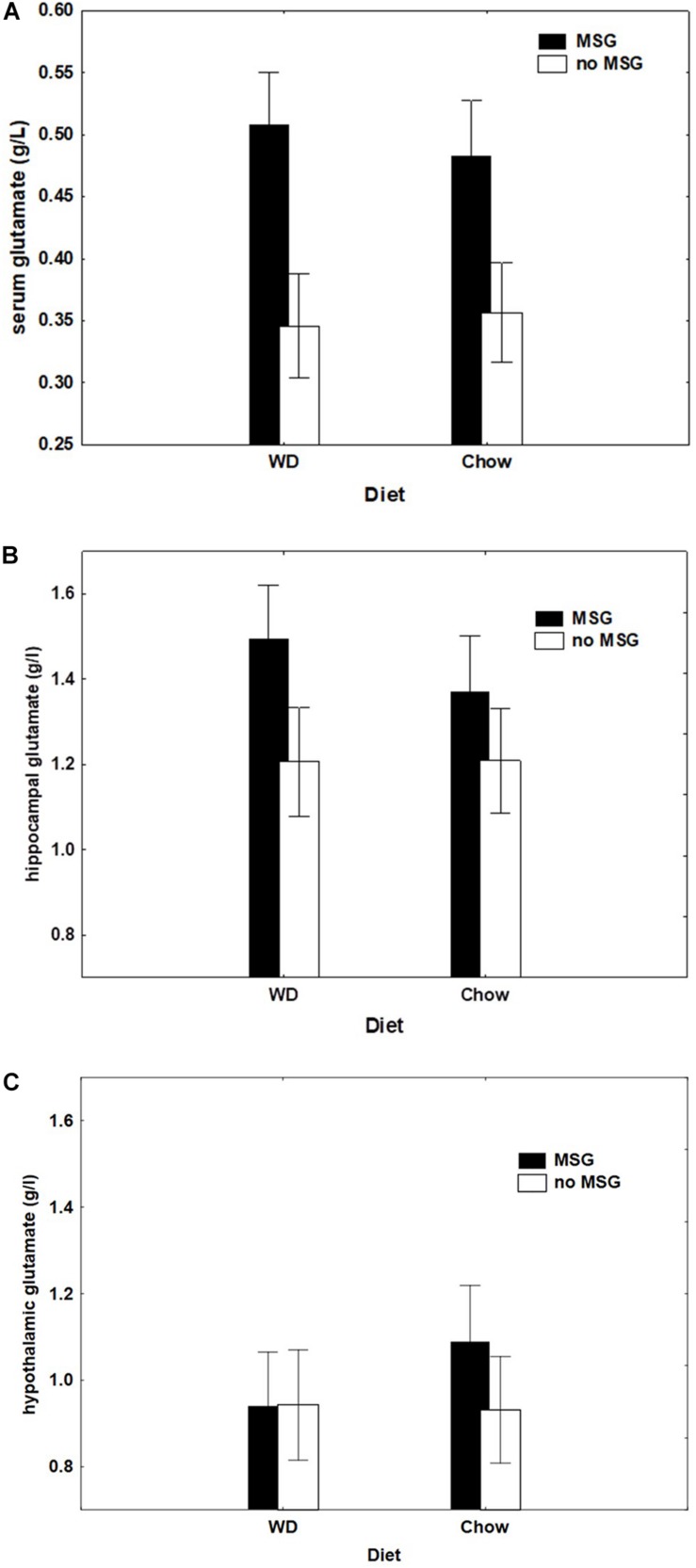
**(A)** Serum glutamate concentration (g/l) as a function of Diet (Chow or WD) and MSG exposure. *N* = 61 animals. **(B)** Hippocampal tissue glutamate concentration (g/l) by diet group (Chow or WD) and MSG exposure. *N* = 61 animals. **(C)** Hypothalamic tissue glutamate concentration (g/l) by diet group (Chow or WD) and MSG exposure. *N* = 61 animals.

Figure 8A shows that rats with MSG added to their test diet exhibited higher levels of serum glutamate independent of whether that diet was WD or Chow. This pattern of results produced a significant main effect of MSG exposure (*p* < 0.01) with no significant main effect of diet or interaction.

Figures 8B,C show hippocampal and hypothalamic brain glutamate levels (respectively) by diet and MSG exposure. Although both groups receiving MSG had higher glutamate levels in the hippocampus, the main effect of MSG fell short of statistical significance (*p* = 0.07), and the MSG by Diet interaction was non-significant. Only the Chow + MSG group appeared to have a higher hypothalamic glutamate concentration; however, here too, neither the main effect of MSG, nor the MSG by Diet interaction achieved significance.

Table 1 shows correlations between the sFN and SD difference scores and serum/brain glutamate concentrations by diet group. No significant correlations were observed for any of the SD difference scores for any group, nor for the sFN difference scores for the WD group. In contrast, the Chow group had the strongest positive correlation (ρ = 0.75, *p* < 0.05) between sFN difference scores and hippocampal glutamate levels, and the WD + MSG group also had a significant positive correlation with hippocampal glutamate levels (ρ = 0.58, *p* < 0.05), which both correspond to the best performance on the sFN test. Conversely, the Chow + MSG group had significant positive correlations between sFN difference scores and both systemic serum glutamate (ρ = 0.64, *p* < 0.05) and hypothalamic glutamate concentrations (ρ = 0.69, *p* < 0.05), while no correlations were observed between sFN difference scores and hippocampal glutamate levels in the Chow + MSG group.

**TABLE 1:** Correlations between brain glutamate concentrations in the hippocampus and hypothalamus and difference scores for serial feature negative (sFN) and simple discrimination (SD) tests by group.

**Variable**	**WD**		**WD + MSG**		**CHOW**		**CHOW + MSG**	
	**sFN diff**	**SD diff**	**sFN diff**	**SD diff**	**sFN diff**	**SD diff**	**sFN diff**	**SD diff**
Serum glutamate	−0.21 NS	0.05 NS	−0.43 NS	−0.26 NS	0.23 NS	−0.01 NS	**0.64 *p* < 0.05**	0.21 NS
Hippocampal glu	−0.07 NS	−0.03 NS	**0.58 *p* < 0.05**	0.20 NS	**0.75 *p* < 0.05**	0.36 NS	0.27 NS	−0.08 NS
Hypothalamus glu	−0.11 NS	0.16 NS	−0.17 NS	−0.21 NS	−0.06 NS	0.03 NS	**0.69 *p* < 0.05**	0.24 NS

*Significant correlations are in boldface.*

## Discussion

This study demonstrates differential effects of MSG on hippocampal-dependent memory performance, with MSG having detrimental effects in the Chow group, but beneficial effects in the WD group. MSG appeared to remedy the well-known negative effects of WD on hippocampal dependent memory performance relative to Chow.

The WD is well known for its ability to impair hippocampal function (Francis and Stevenson, 2013; Kanoski and Davidson, 2011; Yeomans, 2017); thus, the finding that WD impaired hippocampal dependent memory relative to Chow is consistent with previous results. The finding of differential effects of MSG for rats fed WD relative to Chow on the hippocampal-dependent task was surprising. Glutamate is actively transported at the BBB in very limited quantities and its transport is thought to be tightly controlled to protect against glutamate induced excitotoxicity (Smith, 2000). However, based on our findings, prolonged exposure to dietary glutamate (with corresponding higher concentrations of serum glutamate) may be able to raise brain interstitial levels over time, with substantial variability in how much this occurs in each animal. This is consistent with clinical trial data suggesting that a sub-set of humans may be sensitive to dietary glutamate (i.e., appearing to not be protected by the blood–brain barrier, as would be expected) (Holton et al., 2012). Here, we demonstrate differential correlations between hippocampal and hypothalamic glutamate concentrations and hippocampal dependent memory performance between diet groups.

The impairment in hippocampal dependent memory performance exhibited by rats that received Chow + MSG may have been caused by altered glutamatergic neurotransmission in the hippocampus. Dysregulated glutamatergic neurotransmission could have occurred through altered expression of glutamate receptors in the hippocampus, as research has shown that loss of NMDA glutamate receptors in the hippocampus can lead to disrupted learning (Brigman et al., 2010). Interestingly, hypothalamic and serum glutamate levels were positively correlated with the Chow + MSG group’s poor hippocampal dependent memory performance. In 2000, Park et al. (2000) reported that a one-time intraperitoneal injection of MSG or aspartate (which has the ability to stimulate the NMDA glutamate receptor) in adult mice, led to impaired memory retention (based on behavioral response to aversive conditioning) and acute damage to hypothalamic neurons, without damage to hippocampal neurons. However, it should be noted that this study did not distinguish between a memory deficit versus a non-specific deficit in behavioral performance. There is evidence of synaptic innervation from the hypothalamus to the hippocampus in rats. Zhang and Hernandez demonstrated that arginine vasopressin from hypothalamic nuclei are an important source of signaling to the hippocampus and may have a modulatory role on hippocampal excitability (Zhang and Hernandez, 2013). Furthermore, arginine vasopressin-containing neurons in the hypothalamus have been shown to be glutamatergic neurons (Ziegler et al., 2002). Thus, it is possible that altered glutamatergic neurotransmission in the hypothalamus could potentially alter hippocampal function; however, much more work is needed to explore this hypothesis. Techniques such as such as microdialysis, quantification of glutamate enzyme and receptor levels, and use of ^1^H-[^13^C]-nuclear magnetic resonance (NMR) spectroscopy may be useful in answering this question.

As demonstrated in previous research, the WD was associated with a reduction in hippocampal dependent memory performance relative to Chow; however, MSG being added to the WD resulted in improved performance on this task. While we cannot directly address the mechanism for this effect in this study, we hypothesize that this positive effect of glutamate may be related to its ability to be used in the Citric Acid Cycle for energy production in times of low glucose availability. We quickly address the potential for this mechanism below in the hopes of informing future research studies on this topic. The protective effect of glutamate in animals receiving the WD may be related to compensation for reduced glucose transport to the hippocampus in these higher adiposity animals. The Western Diet used in this study has been shown to cause reduced expression of GLUT1 transporters in previous research (Hargrave et al., 2015). The effects of the WD on GLUT1 expression tend to occur quickly (within days of receiving the diet), and thus, may be an early precipitating event which causes hippocampal dysfunction. Interestingly though, this early effect is remedied within a few weeks, and then hippocampal dysfunction appears again later (Hargrave et al., 2015; Jais et al., 2016). This effect may also be caused by insulin resistance in the brain (affecting GLUT4 expression) which can reduce access to glucose during cognitively demanding tasks (Pearson-Leary and McNay, 2016; Grillo et al., 2015). Furthermore, several studies have shown that long-term WD exposure increases BBB permeability that is selective for the hippocampus (see for example Hargrave et al., 2016).

In times of low glucose supply, amino acids can be used to provide necessary metabolites for the TCA cycle to continue to produce ATP (Berg et al., 2002). For glutamate to serve as a substrate, this must happen via allosteric regulation of glutamate dehydrogenase (GDH) (Li et al., 2012). Glutamate dehydrogenase is a reversible enzyme responsible for interchanging glutamate and alpha-ketoglutarate in the TCA cycle (Nissen et al., 2017). During times of high glucose intake (with corresponding higher production of GTP and ATP), alpha-ketoglutarate can be converted into glutamate for use as a neurotransmitter, and as a precursor to glutamine, GABA, and glutathione (Berg et al., 2002). Conversely, in times of glucose shortage, glutamate can be converted into alpha-ketoglutarate to support ATP production (Stanley, 2009). In 2015, Nissen et al. (2015) demonstrated that a reversal of GDH activity in astrocytes appears to increase utilization of amino acids (including glutamate) as substrates for TCA cycle intermediates. Moreover, prior research has shown that giving certain amino acids can rescue cognitive function during glucose deprivation (Evans et al., 2004; Rossetti et al., 2008).

To our knowledge, this is the first study to show that dietary glutamate can impair hippocampal function in animals receiving a typical Chow diet, while conversely also rescuing hippocampal function in higher adiposity animals receiving the WD. One limitation to this research is that we did not include metabolic measures such as fasting glucose and insulin levels, nor did we measure the expression of potentially important glucose transporters such as GLUT1 and GLUT4. Future research is needed to test the hypothesis that the provision of specific amino acids can rescue hippocampal function in animals with diet induced cognitive dysfunction. We recommend that future research test other amino acids (such as the branched chain amino acids) in addition to glutamate, since other amino acids carry less risk of potential adverse effects (e.g., excitotoxicity). Additionally, it is also of interest for future studies to measure both blood glucose and insulin resistance, as well as to examine both GLUT1 and GLUT4 expression concurrently, to identify whether restriction of access to glucose may be occurring, and if so, by which mechanisms.

Finally, it is essential that this study also be repeated using both male and female animals to test for sex differences in future research. Sex differences have been noted in animals receiving neonatal injections of MSG (Clough et al., 1986; Sagae et al., 2011; Rudyk et al., 2018), which has been used for years to induce hypothalamic damage that results in obesity and type II diabetes (Bunyan et al., 1976). Sex differences have also been reported with dietary MSG exposure in mice (Collison et al., 2011). Additionally, sex differences may also be important for the cognitive control of feeding (Sample and Davidson, 2018). Therefore, it is essential that future research on glutamate include both males and females.

## Conclusion

This study demonstrates differing effects of dietary glutamate on hippocampal dependent memory function, with MSG impairing hippocampal function in animals receiving chow, while improving hippocampal function in animals receiving a Western-type diet, high in fat and sugar. More research is needed to explore the mechanism behind these differential effects.

## Data Availability

Datasets are available on request. The raw data supporting the conclusions of this manuscript will be made available by the authors, without undue reservation, to any qualified researcher.

## Ethics Statement

The care and use of all animals in this study was reviewed and approved by the American University Institutional Animal Care and Use Committee.

## Author Contributions

KH and TD contributed to the conception, design of the study, and interpretation of the data. SH collected the data. TD oversaw data acquisition and analysis. All authors participated in the production of the manuscript.

## Disclaimer

This article was prepared while SH was employed at American University. The opinions expressed in this article are the author’s own and do not reflect the view of the National Institutes of Health, the Department of Health and Human Services, or the United States Government.

## Conflict of Interest Statement

The authors declare that the research was conducted in the absence of any commercial or financial relationships that could be construed as a potential conflict of interest.
